# A step toward essential tremor gene discovery: identification of extreme phenotype and screening of *HTRA2* and *ANO3*

**DOI:** 10.1186/s12883-016-0748-3

**Published:** 2016-11-23

**Authors:** Mathilde Renaud, Christophe Marcel, Gabrielle Rudolf, Mickaël Schaeffer, Ouhaïd Lagha-Boukbiza, Jean-Baptiste Chanson, Jamel Chelly, Mathieu Anheim, Christine Tranchant

**Affiliations:** 1 Service of Neurology, University Hospital of Strasbourg, Hospital of Hautepierre, 1 avenue Molière, 67098 Strasbourg Cedex, France; 2 Fédération de Médecine Translationnelle de Strasbourg (FMTS), Université de Strasbourg, Strasbourg, France; 3 Institut de Génétique et de Biologie Moléculaire et Cellulaire (IGBMC), INSERM-U964/CNRS-UMR7104/Université de Strasbourg, 67404 Illkirch, France; 4 Département d’informations médicales, Hôpitaux universitaires de Strasbourg, Hôpital civil, 1 place de l’Hôpital, 67000 Strasbourg, France; 5 Service de Diagnostic génétique, Nouvel hôpital civil, 67000 Strasbourg Cedex, France

**Keywords:** Essential tremor, Electrophysiological recording, Extreme phenotype, *ANO3*, *HTRA2*

## Abstract

**Background:**

Essential tremor (ET) is characterized by a frequent family history. No monogenic form of ET has been identified. We aimed at exploring ET patients to identify distinct subgroups and facilitate the identification of ET genes. We tested for the presence of *HTRA2* p.G399S, and *ANO3* p. W490C, p. R484 W and p. S685G mutations.

**Methods:**

Between June 2011 and November 2013, all consecutive patients suspected with ET were prospectively included in a prospective, monocentric study. Family history, age at onset (AAO), features of tremor, benefit of alcohol and drugs, electrophysiological recording findings were collected. Sanger sequencing was performed for *HTRA2* and *ANO3* mutations screening.

**Results:**

Sixty eight patients were investigated. Fourteen diagnosed with psychogenic (5) or dystonic tremor (9) were excluded. Regarding the 54 ET patients, mean AAO was 48 years (6–77), and mean disease duration 15 years (1–55). Bimodal distribution of AAO was consistent with phenotypic subgroups. In patients with AAO before 30 years, marked benefit of alcohol (*p* < 0.01) and ET family history (*p* < 0.01) were more frequent and the disease progression less severe (*p* < 0.0001). Neither *HTRA2* nor *ANO3* mutation were identified in our patients.

**Conclusions:**

Our data support that distinct ET phenotypic subgroups may be encountered. We recommend to study separately extreme phenotypes of ET, particularly autosomal dominant families with early AAO (<30 years) and marked benefit of alcohol, to facilitate the identification of ET genes. Electromyographic recording remains a support to distinguish ET from differential diagnosis. *HTRA2* and *ANO3* mutations are not common causes of ET.

## Background

According to the Consensus Statement of the Movement Disorder Society (CSMDS) [1], essential tremor (ET) is characterized by: (1) a bilateral, largely symmetric, postural tremor with or without kinetic component, which affects hands and forearms, that is present for at least 5 years, visible and persistent, (2) additional or isolated tremor of the head may occur but in the absence of abnormal posturing, and (3) exclusion of other causes for tremor (other neurological diseases, iatrogenic tremor, exaggerated physiological tremor, recent history of head trauma, psychogenic trigger). Nevertheless, debates continue about the definition of ET because it is an heterogeneous disorder that has no clear diagnostic test [2].

Clinical practice emphasizes ET heterogeneity, and the limits of the above definition: family history is frequent but may be lacking, histogram of age at onset (AAO) demonstrates bimodal distribution with a first peak in childhood or adolescence [3] and a second around the 5th and 6th decades. In the same way, unilateral cases have been described [4], or cases associated with other motor (rest tremor, cerebellar ataxia) or non-motor (depressive syndrome, sleep disorders, cognitive decline) signs have been reported [5], and response to alcohol or to beta-blockers are only optional. Moreover, distinct pathological patterns have been reported [6]: one with cerebellar lesions, loss of Purkinje cells and presence of torpedoes; another with brainstem Lewy bodies.

Such heterogeneity added to the lack of stringent diagnostic criteria or of biomarkers and to the evidence for non mendelian inheritance could explain the weak profitability of genetic studies and their difficulty to find reproducible data [7]. Indeed, a mutation in the *FUS* gene [8] was found in only one ET family. *LINGO1* and *SLC1A2* polymorphisms have been only identified in genome-wide association studies [9–11]. An *HTRA2* variant has very recently been associated in one family with ET and optional parkinsonism [12]. However, a clear, monogenic form of ET is still lacking. *ANO3* mutations were reported in a spectrum of dystonic patients initially presumed to have familial essential tremor [13].

We aimed at analysing a cohort of patients with ET to identify distinct phenotypic subgroups in order to facilitate the identification of genes responsible for ET, and searched for *HTRA2* and *ANO3* mutations recently identified as causes of family ET with extrapyramidal features.

## Methods

From June 2011 to September 2013, all the patients referred in our tertiary movement disorders center with suspected ET, according to the CSMDS [1] definition were included in a prospective, monocentric study. All patients provided informed consent for the study and to be videotaped, and the local ethics committee approved the study.

### Clinical examination

Following items were collected: family history, AAO, current age, clinical features of tremor, severity scales (The Essential Tremor Rating Assessment Scale score (TETRAS) performance subscale [14]), marked benefit of alcohol (patients were asked to answer to the question “does your tremor markedly improve following alcohol consumption”) and to drugs, clinical signs associated to tremor, severity of disease progression (defined by the TETRAS performance subscale/duration of ET ratio). A video recording was performed for each patient, enabling the confirmation of the diagnosis of ET by a second neurologist devoted to movement disorders.

### Electrophysiological recordings

Using (1) surface electrodes on extensor and flexor muscles of both forearms, and on cervical muscles, and (2) an unidirectional piezoelectric accelerometer placed on the index finger of dominant hand, the following items were analysed: the conditions of occurrence of the tremor (rest, posture, intention), frequency, regularity, agonist-antagonist synchronicity, distribution, and distractibility. Accelerometer signal was bandpass filtered at 20–500 Hz. Postural and intention tremor were analyzed during postural maintenance and action (goal-directed movements and drawing). The effect of mental calculation was tested at rest. Patients with atypical tremor including distractible electrophysiological patterns (variations of frequency, transient disappearance of the tremor) suggestive of psychogenic tremor and patients with suspected dystonic tremor (abnormal posture, agonist-antagonist muscles co-contraction) were excluded from the study.

### Biology and Sanger sequencing

Cupper, ceruloplasmin and thyroid stimulating hormone (TSH) serum levels were analysed in each patient. *FMR1* premutation was investigated in all patients with onset after 50 years in order to rule out fragile X-associated tremor ataxia syndrome (FXTAS) [15]. For screening of *HTRA2* p.G399S, and *ANO3* p.W490C, p.R484 W and p.S685G variants recently reported as causes of ET, DNA was extracted from whole blood using illustra™ DNA Extraction kit BACC3 (GE Healthcare UK).. The coding exons of *HTRA2* and *ANO3*, reported recently [12, 13] were screened for mutations by Sanger sequencing of genomic DNA. Fifty-microliter PCR reactions were carried out with 40 ng of genomic DNA and 10pmol of each pair of primer of forward and reverse primers using KAPA Hifi Hotstart ReadyMix PCR Kit (KAPABiosystems, Boston, Masschusetts, United States). Primer pairs are available upon request. All PCR products were purified and both strands were sequenced at GATC Biotech (http//www.gatc-biotech.com/). Sequenced were analysed using the SEQSCAPE v2.5 software (Applied Biosystems, Courtaboeuf, France).

### Brain MRI and DAT-SPECT

Cerebral MRI was performed in 28 cases and, dopamine transporter single-photon emission tomography (DAT-SPECT) was performed in 8 of the 10 patients who also had rest tremor.

### Statistical analysis

Statistical analyses were performed using parametric and non-parametric tests, according to the data distributions. Association between categorical variables was assessed using Fisher-exact tests, because of the small populations in each level of the qualitative variables.

Correlation between quantitative variables has been tested using Pearson correlation tests and Spearman correlation tests, in order to check for linear and non-linear correlation assumption.

Link between a qualitative and a quantitative variable was checked using the non-parametric Kruskal-Wallis test, because of the nature of continuous variables distributions. The Gaussian assumption of these formers was not always checked (Shapiro-Wilk test), implying the use of those non-parametric tests.

Alpha risk was fixed to 5%. Analyses were performed using the R software (version 3.0.2).

## Results

Sixty-eight patients were investigated. Taken together, clinical and neurophysiological findings were finally consistent with psychogenic tremor in 5 patients and dystonic tremor in 9 patients who were excluded.

**Fig. 1:**
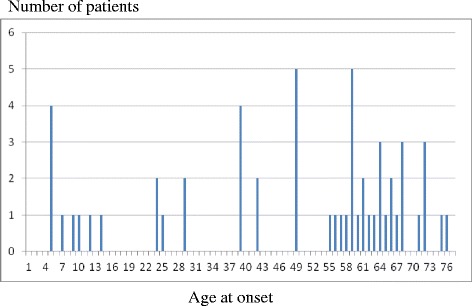
Bimodal distribution of age at onset of tremor

The main clinical and electrophysiological data of the remaining 54 patients are summarized in Tables 1 and 2 and in Fig. 1.

**Table 1:** Demographic, medical and upper limb electrophysiologic data of the cohort

	Patients (*n* = 54)
Demographic features	
Female	25 (46%)
Male	29 (54%)
Mean age at evaluation, range	62 years (18–80)
Mean AAO of tremor, range	48 years (6–77)
Duration of tremor, range	14.5 years (1–55)
Family history of essential tremor	29 (54%)
Family history of Parkinson’s disease	2 (3.5%)
Family history of ataxia	0
Family history of other neurodegenerative disease	2 (3.5%)
Marked benefit of alcohol (*n* = 44 patients)	13 (30%)
Increased at morning^a^	10 (20%)
Increased with emotions^b^	44 (82%)
Increased with physical exercise^c^	22 (41%)
Initial location of tremor	
One hand	27 (50%)
Dominant hand	23 (42%)
Two hands	27 (50%)
Current location of tremor	
Upper limbs	54 (100%)
Bilateral	54 (100%)
Unilateral	0
Symmetrical	17 (32%)
Lower limbs	4 (7%)
Head	14 (26%)
Voice	9 (17%)
Trunk	2 (4%)
Other clinical signs	
Akinesia	5 (9%)
Ataxia	2 (4%)
TETRAS score (performance subscale), range	8.5 (1–21)
Electrophysiologic findings in upper limb extremities	
Rest tremor	10 (18%)
Postural tremor	54 (100%)
Intentional tremor	46 (85%)
Postural tremor only	5 (9%)
Rest, postural and intentional tremor	8 (15%)
Frequency of postural tremor	6.3 Hz (3.8–11)

*TETRAS* The Essential Tremor Rating Assessment Scale; *AAO* age at onset, *Hz* Hertz

^a^amplitude of tremor more important relative to the rest of the day

^b^amplitude of tremor more important with emotions (happiness, sadness)

^c^amplitude of tremor more important with sustained exercise

**Table 2:** Correlation between age at onset and other characteristics of tremor (*n* = 42 patients)

	AAO ≤ 30 years (early onset ET)	AAO ≥ 55 years (late onset ET)	*p*
	(*n* = 14)	(*n* = 28)	
Family history of essential tremor (*n* = 42)	93% (*n* = 13)	36% (*n* = 10)	0.0007
Benefit of alcohol (*n* = 33)	54% (*n* = 6)	9% (*n* = 2)	0.008
Duration of tremor, years (SD) (*n* = 42)	29 (+/−16)	6.5 (+/−6)	<0.0001
TETRAS score/duration of tremor (SD) (*n* = 42)	0.4 (+/−0.2)	2.3 (+/−2.3)	<0.0001
Head tremor (*n* = 42)	21% (*n* = 3)	29% (*n* = 8)	1.00
Lower limbs tremor (*n* = 42)	21% (*n* = 3)	0% (*n* = 0)	0.03
Rest tremor (*n* = 42)	21.4% (*n* = 3)	17.8% (*n* = 5)	0.85
Mean frequency, Hertz (SD) (*n* = 42)	7.0 (+/−2)	6.2 (+/−1)	0.23
TETRAS score (SD) (*n* = 42)	9.6 (+/−4)	7.8 (+/−5)	0.15
Response to beta-blockers (*n* = 23)	77% (*n* = 10)	70% (*n* = 7)	1.00
Response to primidone (*n* = 10)	80% (*n* = 4)	60% (*n* = 3)	1.00

*AAO* age at onset, *SD* standard deviation, *TETRAS* The Essential Tremor Rating Assessment Scale

### Patients

The 54 studied patients had a mean age of 62 years (range, 18 to 80) and had a mean history of tremor of 14.5 years (range, 1 to 55). The mean AAO was 48 years (range, 6 to 77). Tremor age of onset showed a bimodal distribution (Fig. 1). A positive family history of tremor was present in 29 (54%) patients. The mean TETRAS performance subscale was 8.5 (range, 1 to 21). Marked benefit of alcohol was found in 13/44 (30%) patients. Marked benefit of alcohol as well as positive family history of ET were correlated with earlier age of onset (*p* = 0.008 and *p* = 0.0007, respectively). TETRAS score was higher when head tremor was present (*p* < 0.001) but head tremor was positively correlated with neither age of onset nor tremor duration.

### Tremor anatomic distribution and tremor patterns

All patients had bilateral upper extremities tremor, 4 (7%) an associated lower limbs and 14 (26%) an associated head tremor. Mean frequency of tremor was 6.3 Hz (3.8–11) in upper extremities. All patients had postural tremor, 46 patients (85%) an associated intentional tremor and 10 (18%) an associated rest tremor.

### Biological, brain MRI, DAT-SPECT and genetic data

Biological analysis (cupper, ceruloplasmin, TSH) was normal in all patients. All the 18 patients investigated for *FMR1* permutation were negative. No patients had *HTRA2* p.G399S, or *ANO3* p.W490C, p.R484 W and p.S685G variant.

Brain MRI was normal in 13/28 cases, but demonstrated vascular leucopathy in 9, cortical frontal atrophy in 2, incidental meningioma in 2 (a parieto-occipital parasagittal left meningomia (6 cm in diameter) and a frontal right meningomia (2 cm in diameter)), and asymptomatic aneurysm of a cerebral artery in 2 patients.

DAT-SPECT demonstrated the loss of dopaminergic neurons in 4/8 patients with rest tremor.

### Medications

Treatments and efficacy of treatments are summarized in Table 3. None of the patients had received anti-psychotic or anti-emetic drugs. None of the following factors were found predictive of response to main anti-tremor drugs (propranolol, primidone, topiramate): ET family history (*p* = 0.28, *p* = 1.0, *p* = 1.0, respectively); marked benefit of alcohol (*p* = 1.0, *p* = 1.0, *p* = 1.0, respectively); associated rest tremor (*p* = 0.32, *p* = 1.0, *p* = 1.0, respectively); symmetrical tremor (*p* = 0.62, *p* = 01.0, *p* = 1.0, respectively); associated head tremor (*p* = 1.0, *p* = 1.0, *p* = 1.0, respectively); associated lower limbs tremor (*p* = 1.0, *p* = 0.30, *p* = 1.0, respectively); frequency (*p* = 0.67, *p* = 0.73, *p* = 0.65, respectively); TETRAS performance subscale (*p* = 0.97, *p* = 0.35, *p* = 0.65, respectively); evolution speed (ratio TETRAS performance subscale/duration of tremor) (*p* = 0.92, *p* = 0.21, *p* = 0.65, respectively).

**Table 3:** Treatments and efficacy of treatments

Medications	Patients (*n* = 54 patients)
Propranolol (*n* = 31 patients)	
Positive effect of propranolol	24 (77%)
Mean posology, en milligram/day, range	109 (40–300)
Mean length of treatment, months, range	57 (1–360)
Primidone (*n* = 16 patients)	
Positive effect of primidone	13 (81%)
Mean posology, en milligram/day, range	338 (10–750)
Mean length of treatment, months, range	15 (1–60)
Topiramate (*n* = 7 patients)	
Positive effect of topiramate	3 (43%)
Mean posology, en milligram/day, range	75 (25–150)
Mean length of treatment, months, range	11 (1–36)
Chronic high frequency thalamic stimulation	2 (4%)

## Discussion

We aimed at identifying extreme phenotype of ET that could be distinguished from other ET variants in order to find genes responsible for ET which appears to be heterogeneous regarding both clinical and genetic basis. Indeed, the identification of gene causing ET remains an important genetic challenge in the field of movement disorders.

Our study confirmed the bimodal distribution of AAO and the frequent positive family history of ET [16, 17] that support genetic heterogeneity of ET. In the same way, our results further strengthen previous data showing that there is a correlation between a family history of tremor and a younger AAO [18].

We found that early onset of tremor, marked benefit of alcohol consumption and positive family history of ET should be considered as such peculiar phenotype also characterized by a less severe progression of the disease. Thus, in order to identify genes causing ET, we recommend to perform genetic analysis in patients with such extreme phenotype. However, these new findings need to be confirmed in larger series. Benefit of alcohol could be quantitatively assessed [19] and could be underpinned by a peculiar pathophysiological process. A family history is clearly suggestive of inheritance of the disease and in the same way, the youngest the onset, the most probable the inheritance.

For the identification of such peculiar phenotype, electrophysiological recording remains a strong support. Indeed, we confirmed the difficulty in some patients to distinguish ET from differential diagnosis such as dystonic tremor or to some extent psychogenic tremor. Therefore, we also recommend to perform electrophysiological recordings for the selection of patients with genuine ET for future genetic studies.

We found that tremor frequency and head tremor should not be considered as determinant factors for the identification of a peculiar phenotype. In our series, some findings could be explained by the natural history of ET rather than by different phenotypes of ET, since patients with earlier age at onset had longer disease duration. Indeed, an age-associated decrease in tremor frequency (approximately 0.06 to 0.08 Hz/year [20]) has been confirmed in our series, where tremor frequency was negatively correlated with current age (*p* < 0.01) and TETRAS subscale performance (*p* < 0.01). Likewise, head tremor which was present in only 11 patients (26.2%, against 31 to 59.4% in previous series) [3, 21], was only correlated with tremor severity (*p* < 0.001). In the same way, the higher frequency of lower limb tremor in patients with earlier age at onset could be due to the natural history of the disease rather than to a peculiar phenotype. However, this statement remains to be confirmed by further studies.

Rest tremor was present in 18% patients, but in opposite to previous data [22], no correlation was found with neither tremor duration (*p* = 0.31) nor TETRAS subscale performance (*p* = 0.2). Some authors suggested that in patients with severe, old and disseminated ET, rest tremor could be explained by diffusion of the pathologic process to the dopaminergic system. However recent post-mortem studies of a few ET patients with rest tremor did not reveal Lewy body pathology [6], and in our series, only four from 8 patients with rest tremor and who underwent DAT scan, demonstrated dopaminergic denervation.

Nevertheless, epidemiological studies have shown that ET could be a risk factor for Parkinson’s disease (PD) [23]. Additional non motor signs such as anosmia or rapid eye movement behaviour disorder in previous series [5] argue for a link between PD and ET. Whether patients affected with both ET and PD should be considered as suitable for the identification of tremor-causing genes needs to be confirmed in a near future. *HTRA2* p.G399S variant has recently been identified to be a risk factor with overlapping family ET and PD but we did not find this variant in our cohort [12]. In the same way, this variant has been very recently searched for in a large series of ET and was finally considered as not a common cause of ET [24, 25]. We did not find *ANO3* mutations in any of our patient using Sanger sequencing. These data have to be replicated in a larger sample of ET patients.

Finally, our series confirmed the therapeutic efficacy of propranolol and primidone [26], and to lesser extent of topiramate, but did not identify any predictors of positive response for any of these drugs. Main limitation of our study is the relatively small number of patients, compared to previous series [3, 16, 21]. However its originality consists of the theory of extreme phenotypes, of comprehensive clinical and electrophysiological data, and of up-to-date genetic studies.

## Conclusions

Larger cohorts are probably needed to confirm that the factors identified in the present study (early age of onset, marked benefit of alcohol, dominantly inherited family history of ET) correspond to an extreme phenotype of ET. Our results suggest to study separately patients with such peculiar phenotype in order to finally find gene responsible for ET.

## Abbreviations

AAO: Age at onset
DAT-SPECT: Dopamine transporter single-photon emission tomography
ET: Essential tremor
FXTAS: Fragile X-associated tremor ataxia syndrome
Hz: Hertz
PD: Parkinson’s disease
TETRAS: The essential tremor rating assessment scale score
TSH: Thyroid stimulating hormone
